# *FOXP3* deep intronic variant underlying IPEX

**DOI:** 10.70962/jhi.20260021

**Published:** 2026-07-29

**Authors:** Pierre Gaufryau, Marie-Claude Stolzenberg, Nathalie Lambert, Philipp Peters, Iben El Missaoui, Najiba Alioua, Sylvain Hanein, Mohamed Hamici, Patrick Nitschké, Peng Zhang, Jacinta Bustamante, Bénédicte Neven, Capucine Picard, Frédéric Rieux-Laucat, Jérémie Rosain

**Affiliations:** 1 Study Center for Primary Immunodeficiencies, Necker Hospital for Sick Children, Assistance Publique Hôpitaux de Paris, Paris, France; 2 Laboratory of Immunogenetics of Pediatric Autoimmune Diseases, INSERM U1163, Imagine Institute, University of Paris Cité, Paris, France; 3 Université Paris Cité, Institut Imagine, Laboratory of Human Genetics of Infectious Diseases, Necker branch, INSERM UMR 1163, Paris, France; 4 Bioinformatic Platform, Institute of Genetic Diseases, Inserm U1163, Imagine Institute, University of Paris Cité and Structure Fédérative de Recherche Necker, Paris, France; 5 Pediatric Immune-Hematology and Rheumatology Unit, Necker Hospital for Sick Children, Assistance Publique Hôpitaux de Paris, Paris, France; 6 Laboratory of Lymphocyte Activation and Susceptibility to EBV Infection, Inserm U1163, Imagine Institute, Paris, France; 7 Laboratory of Human Genetics of Infectious Diseases, University of Texas Southwestern Medical Center, Dallas, TX, USA; 8 Center for the Genetics of Host Defense, University of Texas Southwestern Medical Center, Dallas, TX, USA; 9 Lyda Hill Department of Bioinformatics, University of Texas Southwestern Medical Center, Dallas, TX, USA

## Abstract

We report a deep intronic hemizygous *FOXP3 *likely pathogenic variant (c.968-207A>G) in a male patient with IPEX that was investigated by RNA sequencing in heterozygous female carriers.

## Teaser

We report a deep intronic FOXP3 pathogenic variant that was investigated by RNA sequencing in heterozygous female carriers.

The global expansion of genomic medicine has improved access to genetic testing for patients with suspected genetic disorders, including inborn errors of immunity (IEI). Despite these advances, overall diagnostic yields in IEI remain modest, with fewer than 50% of patients receiving a definitive molecular diagnosis. One major limitation is the predominant focus of genetic testing on protein-coding regions and essential splice sites. In addition, high-throughput sequencing (HTS) approaches that capture mainly exons (i.e., targeted gene panels or whole-exome sequencing) limit variant detection outside these regions. Consequently, variants located in noncoding regions are frequently overlooked (1, 2). Recently, several in silico tools have been developed to screen variants in noncoding regions, particularly intronic variants predicted to affect splicing, including SpliceAI and Splicing Prediction Pipeline (SPiP). In parallel, RNA-based analyses to characterize the functional consequences of such variants are increasingly implemented in clinical laboratories, in accordance with current guidelines (3). Deep intronic variants may impair splicing by creating or activating cryptic splice sites, by disrupting branch points, or through other mechanisms. These alterations can result in pseudo-exon inclusion, exon skipping, or transcript destabilization.

**Figure 1.**
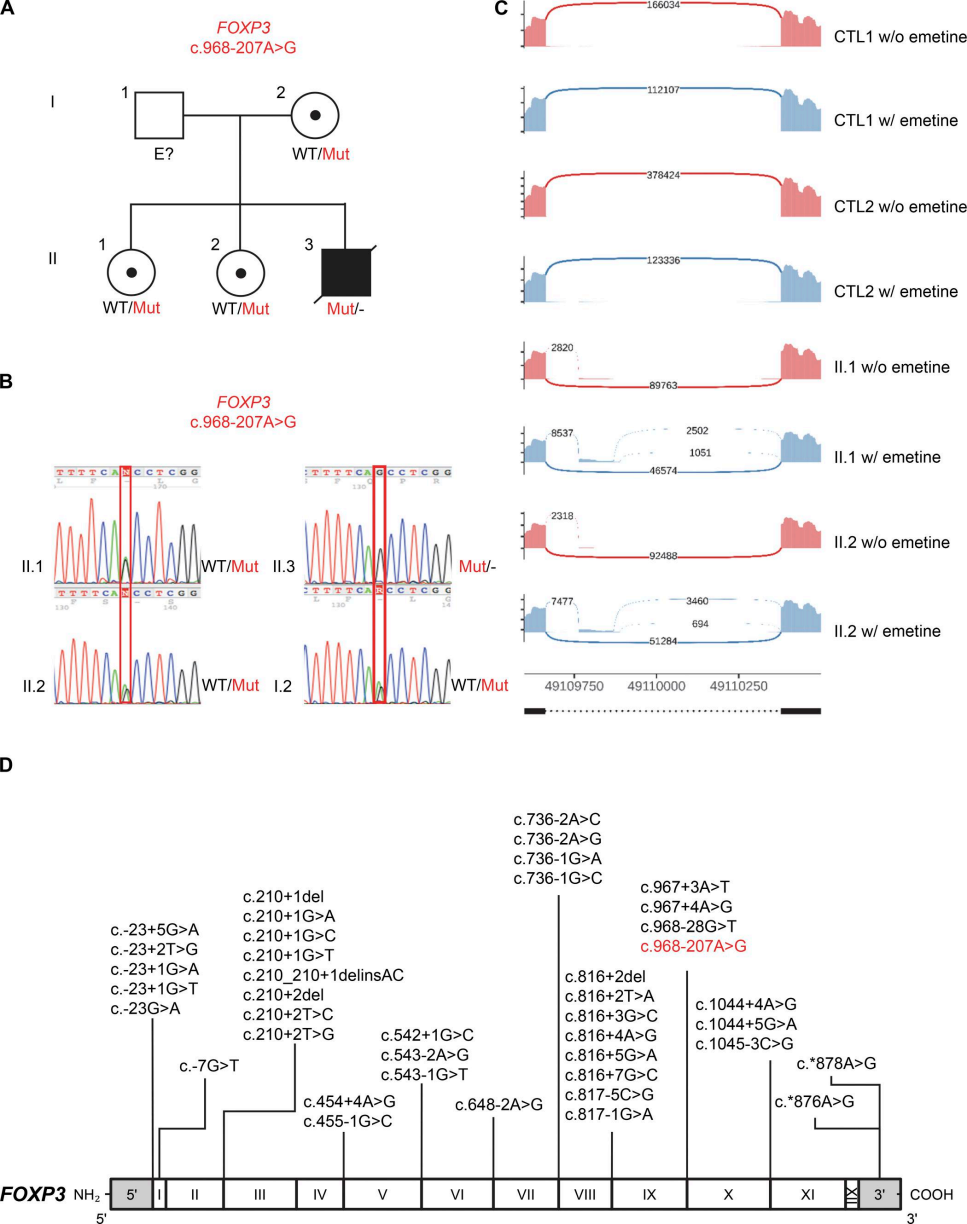
**Single-nucleotide deep intronic variant underlying IPEX. (A)** Pedigree tree. Male and female individuals are represented by squares and circles, respectively. Each generation is designated by a Roman numeral and each individual by an Arabic numeral. Individuals with immune dysregulation are shown as closed black symbols. Black dots represent heterozygosity. Mut = mutated; WT = wild type; E? = unknown genetic status **(B)** Electropherograms for the sequencing of representative *FOXP3* nucleotide sequences present at the hemizygous state in the patient, and at the heterozygous state in his mother and sisters. **(C)** Sashimi view of *FOXP3*-targeted RNA sequencing from *FOXP3* PCR performed on cDNA extracted from activated T cells from two healthy controls (CTLs) and the two sisters, in the presence (w/) and absence (w/o) of emetine. **(D)** Review of the literature of pathogenic or likely pathogenic *FOXP3 *variants located outside the coding sequence.

We investigated a male patient born to nonconsanguineous French parents (Fig. 1 A). Both parents were healthy, and the mother had no history of miscarriage. The patient had two older sisters, both of whom were healthy. He was born at term in 1998 following an uncomplicated pregnancy. The patient developed multiple early-onset autoimmune and atopic manifestations. Eczema was present from three weeks of age. At nine months old (mo) of age, he was diagnosed with severe autoimmune enteropathy with villous atrophy and positive anti-AIE75 autoantibodies. At 13 mo of age, he developed Graves’ disease with positive thyrotropin receptor autoantibodies. At six years of age, he presented with immune thrombocytopenia associated with positive anti-platelet (2B3A-type) autoantibodies. He also exhibited multiple food and latex allergies. Immunological evaluation revealed fluctuating mild hypereosinophilia and chronic severe hyper-IgE (>10,000 kUI/L). The following phenotyping was performed when the patient was 6 years old, and thus found to have normal proportion of T and B cells, without excess of activated T cells: CD3^+^ = 83% (*N* = 56–75); CD3^+^CD4^+^ = 62% (*N* = 28–47); CD3^+^CD8^+^ = 21% (*N* = 16–30); CD19^+^ = 12% (*N* = 6–25); CD3^+^HLADR^+^ = 6% (*N* < 15%); CD3^+^CD4^+^HLADR^+^ = 6% (*N* < 15%); CD3^+^CD8^+^HLADR^+^ = 3% (*N* < 15%). The patient received multiple immunosuppressive treatments, including prednisone, tacrolimus, sirolimus, and rituximab. He underwent hematopoietic stem cell transplantation (HSCT) at 7 years of age from an HLA-identical sister donor. He died 47 days after HSCT due to severe graft-versus-host disease. Overall, the clinical presentation of the patient was highly suggestive of X-linked immunodysregulation, polyendocrinopathy, enteropathy syndrome.

In 2005, the patient underwent Sanger sequencing of *FOXP3* using primers covering the coding sequence and essential splice sites. No pathogenic variant was identified. Genetic investigations were resumed in the 2020s during genetic counseling of the two sisters. However, insufficient DNA material remained from the index case to perform HTS. Both sisters were analyzed using a targeted gene panel covering 331 known IEI genes. No candidate single-nucleotide variants or copy-number variants were detected in the coding regions or essential splice sites of the captured genes, including *FOXP3*. The probes of the panel are designed to capture exons, but flanking introns are also partially covered (1). We thus also analyzed variants in covered introns and found that both sisters carried a heterozygous intronic variant in intron 9 of *FOXP3* (NM_014009.4:c.968-207A>G; hg38:X-49253409-T>C), of which the genomic region was captured by the probes. The sequencing depth for this position was 59X and 11X, in II.1 and II.2, respectively. Its presence was confirmed by Sanger sequencing at the heterozygous state in both sisters and in their mother, and at the hemizygous state in the patient (Fig. 1 B). The variant is extremely rare, as it is absent from public population databases, including gnomAD v4.1.0. The CADD v1.7 score for the variant was 6.32. The variant was not predicted to lie within a regulatory element according to Regulatory Mendelian Mutation or PromoterAI scores, nor was it located at a conserved nucleotide position based on PhyloP (score = 0.044). In contrast, the c.968-207A>G variant was predicted to impair splicing by SpliceAI (maximum score = 0.67), Pangolin (score = 0.2), and MaxEntScan (350% increase in splice site strength), but not by SPiP. Overall, we identified a rare deep intronic hemizygous *FOXP3 *variant predicted to impair splicing through the creation of a novel acceptor site.

To assess the pathogenicity of this variant, RNA studies were performed (4). As no biological material was available from the index case, peripheral whole blood samples from the two carrier sisters were used. CD3/CD28-activated T cells were generated from both sisters and from healthy controls. After 6 days of culture, T cells were treated overnight with or without emetine, which is an inhibitor of nonsense-mediated mRNA decay used to stabilize aberrant transcripts (4). RNA was extracted, and a full-length *FOXP3* cDNA PCR was performed generating a 1,212-bp-length amplicon (using the following primers: forward 5′-ATG​CCC​AAC​CCC​AGG​CCT-3′ and reverse 5′-TCA​GGG​GCC​AGG​TGT​AGG​G-3′). The resulting amplicon was sequenced using targeted short-read HTS with a mean coverage of 100,000X. Splice junction usage was analyzed using Sashimi plots with a threshold excluding alternative splice representing <5%. In both sisters, but not in healthy controls, additional 106-bp (chrX:49,109,764-49,109,869; *FOXP3* (NM_014009.4):r.967_968ins106 p.(Gln323fs*35)) and 125-bp (chrX:49,109,764-49,109,888; *FOXP3* (NM_014009.4):r.967_968ins125 p.(Gln323fs*22)) two exons were detected between exons 9 and 10, confirming the predicted creation of alternative acceptor sites and activation of cryptic donor sites (Fig. 1 C). In the presence of emetine, these aberrant transcripts accounted for 16 and 13% of reads in the two sisters, respectively, relative to the wild-type transcript (Fig. 1 C). In the absence of emetine, these aberrant transcripts accounted for 3 and 2% of reads in the two sisters, respectively, relative to the wild-type transcript. According to American College of Medical Genetics and Genomics criteria, these data support classification of the c.968-207A>G variant as likely pathogenic and causative of the patient’s clinical phenotype.

We report the first deep intronic variant identified in *FOXP3*. Although several splice-altering *FOXP3* variants have previously been described (5), these were located in close proximity to exon–intron boundaries (Fig. 1 D). The most distal reported variants affected intron 8, at five bp upstream of the acceptor site (c.817-5C>G) and seven bp downstream of the donor site (c.816+7G>C) (5). Such variants should be systematically considered in patients with unexplained phenotypes, particularly through whole-genome sequencing combined with dedicated splice prediction tools. The use of multiple prediction algorithms is essential, as illustrated here by discordant predictions between MaxEntScan and SpliceAI versus SPiP. In this study, T cell activation enhanced FOXP3 expression compared with peripheral blood mononuclear cells at the resting state (unpublished data), thereby increasing sensitivity for detection of aberrant transcripts. In addition, emetine treatment unmasked splice-altered transcripts that would otherwise remain undetectable in heterozygous female carriers. Alternatively, the variant could have been investigated using overexpression systems, such as minigene or exon-trapping assays (4), which have not been performed here and constitute a limitation of our study to firmly establish that the likely pathogenic variant c.968-207A>G is pathogenic. RNA analysis from formalin-fixed paraffin-embedded tissues may also provide complementary evidence for the pathogenicity of deep intronic variants. Overall, these findings further highlight the importance of investigating noncoding variants in patients with suspected IEI.

## Ethics approval

Informed consent for participation in this study was obtained in accordance with local regulations, with approval from the Institutional Review Board in France.

## Consent to participate

Written informed consent to participate was obtained from his siblings or his parents.

## Consent for publication

Consent to publish this report was obtained from his siblings or his parents. All the authors approved the final version of the manuscript.

## Data Availability

All data are either included in the manuscript or available upon request.
